# Comprehensive analysis of lncRNA biomarkers in kidney renal clear cell carcinoma by lncRNA-mediated ceRNA network

**DOI:** 10.1371/journal.pone.0252452

**Published:** 2021-06-08

**Authors:** Ke Gong, Ting Xie, Yong Luo, Hui Guo, Jinlan Chen, Zhiping Tan, Yifeng Yang, Li Xie

**Affiliations:** 1 Department of Cardiovascular Surgery, The Second Xiangya Hospital of Central South University, Central South University, Changsha, P.R. China; 2 The Clinical Center for Gene Diagnosis and Therapy of The State Key Laboratory of Medical Genetics, The Second Xiangya Hospital of Central South University, Central South University, Changsha, Hunan, P.R. China; University of Science and Technology Liaoning, CHINA

## Abstract

**Introduction:**

Kidney renal clear cell carcinoma (KIRC) has a high incidence globally, and its pathogenesis remains unclear. Long non-coding RNA (lncRNA), as a molecular sponge, participates in the regulation of competitive endogenous RNA (ceRNA). We aimed to construct a ceRNA network and screened out possible lncRNAs to predict KIRC prognosis.

**Material and methods:**

All KIRC data were downloaded from the TCGA database and screened to find the possible target lncRNA; a ceRNA network was designed. Next, GO functional enrichment and KEGG pathway of differentially expressed mRNA related to lncRNA were performed. We used Kaplan-Meier curve analysis to predict the survival of these RNAs. We used Cox regression analysis to construct a model to predict KIRC prognosis.

**Results:**

In the KIRC datasets, 1457 lncRNA, 54 miRNA and 2307 mRNA were screened out. The constructed ceRNA network contained 81 lncRNAs, nine miRNAs, and 17 mRNAs differentially expressed in KIRC. Survival analysis of all differentially expressed RNAs showed that 21 lncRNAs, four miRNAs, and two mRNAs were related to the overall survival rate. Cox regression analysis was performed again, and we found that eight lncRNAs were related to prognosis and used to construct predictive models. Three lnRNAs from independent samples were meaningful.

**Conclusion:**

The construction of ceRNA network was involved in the process and transfer of KIRC, and three lncRNAs may be potential targets for predicting KIRC prognosis.

## Introduction

Renal cell carcinoma (RCC) is a predominant form of cancer in humans, 3% of all cancers are RCC, with the highest incidence occurring in Western countries. Over the past 20 years, the global and European morbidity rate has increased at a rate of 2% per year. There are an estimated 99,200 new RCC patients in the European region and 39,100 deaths from kidney cancer in 2018 [1]. The incidence of renal clear cell carcinoma (KIRC) is very high, accounting for 80–90% of renal malignancies [2]. Although detection methods have improved with the development of ultrasound and computed tomography, many kidney lumps remain asymptomatic and will not be discovered until late in the disease. Compared with other RCC types, KIRC has a high recurrence and metastasis rate. Moreover, the 5-year survival rate of metastatic patients with KIRC is less than 10% [3]. KIRC also has few biomarkers. Therefore, the detection and identification of new and sensitive biomarkers are essential for improved prediction of progress and prognosis of metastatic KIRC.

Long non-coding RNA (lncRNA) is a subset of transcripts with a length of more than 200 non-protein coding nucleotides [4]. LncRNA occupies a large proportion of ncRNA. They have increasingly been related to many important biological processes, especially lncRNAs. They play important roles in chromatin modification, cell differentiation and proliferation, RNA processes, apoptosis and human diseases [5–9]. MiRNAs are highly conserved and interact with target mRNA to silence genes. In gene expression, other RNA transcripts are regulated by competing for endogenous RNA (ceRNA) through competition for shared miRNAs. As lncRNAs and mRNAs share a common miRNA response element, the relationship network of lncRNA-miRNA-mRNA is essential for regulating RNA expression. Furthermore, lncRNAs function as “molecular sponges” that directly or indirectly competitively binds to miRNA, eventually weakening the effect of miRNAs on mRNA [10–13].

The ceRNA network plays a regulatory role in many cancers of gastric cancers [14, 15], including pancreatic adenocarcinoma [16, 17], lung cancer [17–19] and colorectal cancer [20, 21]. In KIRC, the Cancer Genome Atlas (TCGA) database has been used to prove that several ceRNAs are significantly related to the overall KIRC survival rate. These studies analyzed the KIRC expression data in the TCGA database, screened out differential genes and constructed a ceRNA network, and used their clinical data to perform survival analysis on the key genes screened out. Their analysis results proved that the differential expression of the selected influencing factors impacts the long-term prognosis of patients [22, 23]. Patients with ccRCC with no PTENP1 expression also have a lower survival rate. PTENP1 acts as a competitive endogenous RNA (ceRNA) in ccRCC, thereby inhibiting cancer development [24]. However, they only obtained the prognostic ceRNA and did not study the construction of prediction models in their research. Here, we first screened all differentially expressed lncRNA (DElncRNA), differentially expressed miRNA (DEmiRNA) and differentially expressed mRNA (DEmRNA), and used them to construct a ceRNA network. Based on this, we screened eight possible lncRNAs as prognostic biomarkers for KIRC through survival analysis and COX regression analysis. Additionally, we established and validated a predictive model of KIRC prognosis based on the lncRNAs. The established model may be a potential method to predict the prognosis of KIRC.

## Materials and methods

### TCGA data collection

RNA sequence data and clinical information were obtained from GDC data portal screening (https://portal.gdc.cancer.gov/) to perform an integrated analysis using the Data Transfer Tool (provided by GDC Apps) according to the published guidelines provided by TCGA (https://www.cancer.gov/about-nci/organization/ccg/research/structural-genomics/tcga) [25]. TCGA is a landmark cancer genome project with molecular characteristics of more than 20,000 primary cancers and matched normal samples covering 33 cancer types. The data retrieved from these databases help us improve diagnostic levels and cancer treatment. Our current research was based on the requirements of the guidelines proposed in the TCGA database. Since RNA sequencing data was obtained directly from TCGA, the approval of the ethics committee was not required. All original data are in S1 File.

### RNA sequence data retrieval and screening of differentially expressed mRNA, miRNA, and lncRNA in KIRC

We used Perl (https://www.perl.org/) and R (https://www.r-project.org/) to analyze and process RNA sequencing data [26, 27]. All the Source codes used are in the S2 File. We merged data from tumor and normal samples through Perl software for subsequent analysis. We collected 611 samples of RNA sequencing data (including 539 KIRC tumor tissues and 72 normal tissues) and 616 samples of miRNA sequencing data (including 545 KIRC tumor tissues and 71 normal tissues; Table 1). To screen for DEmRNA, DEmiRNA, and DElncRNA, we used the “edgeR” package of R software to compare KIRC with normal samples. |log2FC|>2 and P-value < 0.01 was the cut-off standard we determined. We used the “gplots” package to draw the volcano and heat maps of the obtained DEmRNA, DEmiRNA, and DElncRNA.

**Table 1:** Characteristics and distribution of patients with kidney clear cell carcinoma in the TCGA-KIRC cohort.

Variables		mRNA-lncRNA (n = 530)	miRNA (n = 516)
age	<65	245	326
	≥65	285	190
gender	male	344	335
	female	186	181
M	M0	420	406
	M1	78	78
	MX	30	30
	NA	2	2
N	N0	239	228
	N1	16	17
	NX	275	271
T	T1	271	259
	T2	69	67
	T3	179	179
	T4	11	11
stage	stage I	265	253
	stage II	57	55
	stage III	123	123
	stage IV	82	82
	NA	3	3

NA: Not available.

### Correlation analysis of lncRNAs, miRNAs, and mRNAs and construction of the ceRNA network in KIRC

To study the relationship between different RNAs, we used them to assemble a ceRNA network in KIRC. The ceRNA network was based on the theory that ceRNA combined with miRNA through response elements to affect mRNA gene expression. We first used the starBase database to convert the miRNA format. Then we used Perl software to explore DEmiRNA interacting with DElncRNA in the miRcode database (http://www.mircode.org/). Next, miRTarBase (http://mirtarbase.mbc.nctu.edu.tw/php/index.php), miRDB (http://www.mirdb.org/) and TargetScan (http://www.targetscan.org/vert_72/) databases were performed to obtain miRNA targeted mRNAs by Perl software with the criteria for each target gene to appear in three different databases [28–30]. Through the “VennDiagrams” package in R software, the predicted miRNA-target genes and DEmRNA were intersected. Based on the selected miRNA-target genes and the determined lncRNA-miRNA relationship, We was assembled ceRNA network to improve the visual analysis of results. Simultaneously, we used Cytoscape (http://www.cytoscape.org/, verson4.0.2) to visualize the results [31]. All figures were typeset using Microsoft PowerPoint software (https://www.microsoft.com/zh-cn/microsoft-365/powerpoint).

### Survival analysis and construction of lncRNA related predictive model

We once again found the patient’s clinical information from the database. The long-term survival of patients with KIRC was analyzed by Kaplan-Meier curve analysis. We used the "survival" software package of R software to analyze the RNA in the ceRNA network. The established P<0.01. We subsequently selected some lncRNAs related to overall survival (P<0.001) according to established criteria as prognostic lncRNA signature candidates for multivariate Cox regression analysis [32]. According to the median risk assessment, patients with KIRC were classified into two cohorts. Receiver operating characteristics (ROC) were used to predict the impact of lncRNA signals (high risk and low risk) on survival [33]. Subsequently, we drew the ROC curve by calculating the area under the curve (AUC) under the binomial exact confidence interval [34].

### Functional enrichment analysis of differentially expressed mRNAs in KIRC

To clarify the gene functions, the function enrichment of DEmRNA related to lncRNA in ceRNA network was analyzed by Gene Ontology (GO) and Kyoto Encyclopedia of Genes and Genomes (KEGG) using the Database for Annotation, Visualization, and Integrated Discovery (DAVID) database (https://david.ncifcrf.gov/home.jsp) and the KOBAS 3.0 online tool (http://kobas.cbi.pku.edu.cn/kobas3) [35, 36]. Additionally, the GOCircle, GOChord, and GOCluster drawing functions of the "GOplot" of R software package were used for expression and functional enrichment analyses [37].

### Reconstruction of the ceRNA sub-network of prognostic in KIRC

To clarify the regulatory mechanism between the potential prognosis of lncRNA and related miRNA and mRNA, we used Cytoscape to extract RNA with prognosis differential to construct a ceRNA sub-network. We also used the Sankey diagram constructed by the “ggalluival” package for visual analysis. All RNA involved were related to the potential prognosis of lncRNA.

### Validation of independent data sets

We selected an independent data set from the Gene Expression Omnibus (GEO) database to verify the results. GSE96574 was used by us for verification. We used Graphpad Prism8 software for statistical analysis and data visualization.

### Statistical analysis

All data were expressed as the means± standard deviation (SD). GraphPad Prism 8.0 (GraphPad Software, San Diego, CA, USA) and R software were used for all statistical analyses. Two-tailed Student’s t-test was used to assess the significance of differences between the two groups. The survival analysis results were obtained from the “survival” package. Cox regression analysis was used to screen lncRNA related to prognosis in the ceRNA network and can be used as a target to predict KIRC prognosis. Unless otherwise stated, statistical significance was set at P<0.05.

## Results

### Screening of differentially expressed RNAs in KIRC

**Fig 1:**
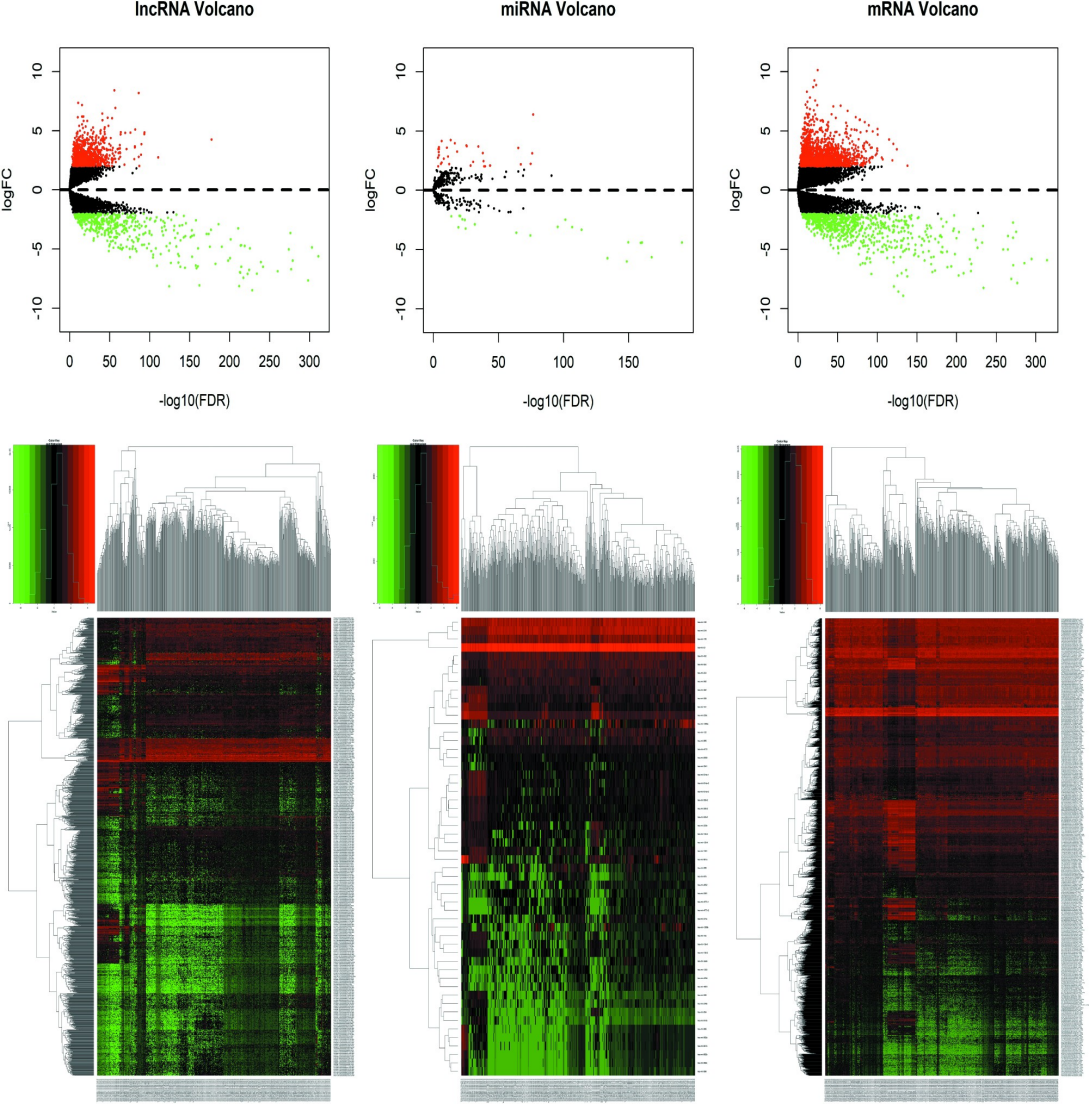
Volcano and heat maps of all differentially expressed RNA between KIRC and normal tissues. (a) Volcano map of DElncRNA in KIRC. (b) Volcano map of DEmiRNA in KIRC. (c) Volcano map of DEmRNA in KIRC. (d) Hierarchical clustering heat map of DElncRNA in KIRC. (e) Hierarchical clustering heat map of DEmiRNA in KIRC. (f) Hierarchical clustering heat map of DEmRNA in KIRC. FDR: False discovery rate.

According to the screening conditions (|log2FC| > 2, P-value < 0.01), 2307 DEmRNAs, 1457 DElncRNAs, and 54 DEmiRNAs were screened out. Tables 2–4 showed the top 20 DElncRNAs, DEmiRNAs, and DEmRNAs. A volcano map (Fig 1A–1C) was drawn to show the distribution of DElcnRNA, DEmiRNA, and DEmRNA in the -log10 (FDR) and logFC dimensions. The hierarchical cluster analysis heat map showed all differentially expressed RNA between KIRC tissues and normal tissues (Fig 1D–1F).

**Table 2:** Top 20 upregulated and downregulated lncRNA in patients with KIRC.

lncRNA	logFC	PValue	FDR
AP005432.2	-6.757905074	0	0
AC019080.1	-3.886047663	0	0
LINC01762	-4.874338157	4.24E-307	9.35E-304
AC090709.1	-7.65872183	3.27E-302	5.77E-299
AP000696.1	-5.997594408	8.83E-283	1.30E-279
AC009035.1	-6.713927435	6.55E-281	8.26E-278
FAM242C	-3.661469455	5.56E-280	6.13E-277
AC068631.1	-5.033045794	6.01E-278	5.90E-275
AC023421.1	-6.883709775	8.55E-264	7.55E-261
LINC01378	-6.507363007	1.51E-245	1.21E-242
AC007993.2	-6.120512627	7.66E-243	5.63E-240
AC073172.1	-7.080454189	1.02E-237	6.90E-235
AC079310.1	-8.482248422	6.71E-232	4.23E-229
AC105384.1	-4.959173524	4.96E-229	2.92E-226
AL139280.1	-6.772062579	5.11E-228	2.82E-225
LINC01571	-7.099142297	4.74E-226	2.46E-223
LINC02410	-4.751856083	2.42E-222	1.19E-219
AC124017.1	-7.440808148	1.14E-220	5.28E-218
AC006441.4	-4.676557282	5.10E-218	2.25E-215
LINC01020	-6.506384384	6.96E-218	2.93E-215
PVT1	4.238882075	1.31E-180	3.51E-178
SLC16A1-AS1	2.729511989	1.56E-113	1.62E-111
AC010655.2	4.842063622	3.92E-96	3.17E-94
AL391845.2	4.704856417	6.04E-96	4.84E-94
CDKN2B-AS1	3.856453462	2.86E-92	2.12E-90
MIR210HG	2.956237343	6.64E-92	4.89E-90
WAKMAR2	2.004757779	1.31E-89	9.12E-88
DARS-AS1	2.770230091	6.90E-89	4.68E-87
TTC21B-AS1	8.176350088	6.94E-89	4.68E-87
LINC02048	5.09657003	2.15E-84	1.33E-82
DGCR9	3.577728993	5.00E-84	3.00E-82
GAS6-AS1	3.668197986	6.34E-84	3.76E-82
SAP30-DT	2.598462928	3.25E-83	1.89E-81
AC137834.2	4.833231799	2.41E-78	1.25E-76
AC026369.2	3.439940746	1.27E-73	5.88E-72
LINC00887	4.812455488	8.95E-72	4.01E-70
AC138207.5	2.110114795	9.00E-71	3.87E-69
AC079760.2	4.868343482	3.36E-66	1.32E-64
SFTA1P	4.67347273	4.40E-66	1.71E-64
AC019069.1	3.108894249	2.01E-65	7.72E-64

FC: Fold change, FDR: False discovery rate.

**Table 3:** Top 20 upregulated and downregulated mRNA in patients with KIRC.

mRNA	logFC	PValue	FDR
TMEM238L	-7.078308523	0	0
ACP3	-5.566157769	0	0
MFSD4A	-5.237894745	0	0
SIM2	-4.49889604	0	0
GPC5	-5.82678567	5.90E-299	1.77E-295
KCNJ10	-6.175499235	6.69E-290	1.72E-286
ELF5	-7.853566134	7.87E-281	1.77E-277
ADGRF3	-3.527227811	2.91E-279	5.83E-276
DDN	-6.340093857	1.57E-275	2.83E-272
ATP1A1	-2.749741864	3.95E-275	6.16E-272
MYLK3	-4.024276299	4.10E-275	6.16E-272
CALB1	-7.504477557	4.96E-273	6.88E-270
HSPA2	-3.886122862	1.02E-268	1.32E-265
IRX2	-5.021990125	1.66E-268	1.99E-265
ENPP6	-5.01847797	1.34E-262	1.51E-259
SLC12A1	-8.26530607	8.10E-238	8.58E-235
MTURN	-3.027205604	2.49E-237	2.49E-234
SLC4A11	-4.437954149	5.85E-236	5.54E-233
TRPV6	-4.864989485	2.91E-230	2.50E-227
FGF1	-4.201610742	5.05E-228	4.13E-225
DDB2	2.049347708	3.57E-141	6.77E-139
NOL3	3.444266195	1.49E-127	2.01E-125
SPAG4	3.90578212	4.21E-123	5.02E-121
SAP30	2.503406987	7.80E-122	9.07E-120
EGLN3	4.276239797	9.80E-118	1.07E-115
VIM	2.593518266	1.77E-109	1.63E-107
ARHGEF39	2.742244652	2.85E-108	2.54E-106
HILPDA	4.690335522	3.29E-103	2.63E-101
GABRD	5.150018388	4.66E-103	3.68E-101
NETO2	3.327264726	1.16E-102	9.11E-101
VEGFA	3.503766201	2.35E-102	1.83E-100
SLC16A3	3.061709145	4.47E-102	3.44E-100
LILRB1	2.942896587	1.38E-100	1.05E-98
CDKN2A	4.947667844	7.98E-97	5.55E-95
ARHGAP22	2.776844532	9.79E-97	6.79E-95
AGAP2	2.717081495	1.43E-96	9.84E-95
CXCR4	2.800644677	7.02E-96	4.81E-94
ST8SIA4	3.592159173	7.67E-96	5.23E-94
COL5A3	3.566548436	3.06E-94	2.01E-92
P2RX7	2.699430547	5.84E-94	3.79E-92

FC: Fold change, FDR: False discovery rate.

**Table 4:** Top 20 upregulated and downregulated miRNA in patients with KIRC.

miRNA	logFC	PValue	FDR
hsa-mir-508	-4.413080209	1.66E-194	7.97E-192
hsa-mir-506	-5.661839583	6.75E-171	1.62E-168
hsa-mir-514a-1	-4.423272889	2.21E-163	3.53E-161
hsa-mir-514a-3	-4.448579466	3.71E-162	4.44E-160
hsa-mir-514a-2	-4.392090737	1.57E-152	1.50E-150
hsa-mir-514b	-6.012465545	2.85E-151	2.28E-149
hsa-mir-934	-5.744736952	1.84E-136	1.26E-134
hsa-mir-509-3	-3.348871994	1.86E-116	1.11E-114
hsa-mir-509-2	-3.116798109	3.85E-109	2.05E-107
hsa-mir-362	-2.4997835	5.73E-104	2.75E-102
hsa-mir-509-1	-3.099261399	5.61E-98	2.44E-96
hsa-mir-129-1	-3.810982115	7.60E-77	2.27E-75
hsa-mir-129-2	-3.582430837	6.41E-66	1.33E-64
hsa-mir-200c	-2.886127557	1.44E-33	1.27E-32
hsa-mir-216b	-3.245682342	2.16E-26	1.46E-25
hsa-mir-203b	-2.51253395	1.07E-25	7.04E-25
hsa-mir-138-1	-2.446678631	8.94E-24	5.49E-23
hsa-mir-138-2	-2.188284603	4.19E-22	2.30E-21
hsa-mir-1251	-2.16487151	1.61E-21	8.57E-21
hsa-mir-184	-3.137223909	8.80E-21	4.44E-20
hsa-mir-122	6.375970805	4.90E-79	1.80E-77
hsa-mir-210	3.106323197	2.65E-78	9.06E-77
hsa-mir-21	2.214057836	5.29E-77	1.69E-75
hsa-mir-584	2.175477131	2.38E-69	5.70E-68
hsa-mir-155	3.563706474	2.20E-67	4.79E-66
hsa-mir-4772	2.073804082	1.60E-45	2.40E-44
hsa-mir-452	2.014972583	1.38E-41	1.94E-40
hsa-mir-142	2.019440538	7.28E-41	9.97E-40
hsa-mir-224	2.453810789	1.72E-40	2.29E-39
hsa-mir-592	3.12806078	7.20E-40	9.33E-39
hsa-mir-885	3.687124949	2.37E-36	2.42E-35
hsa-mir-3941	2.377116049	2.76E-32	2.32E-31
hsa-mir-6509	2.080279385	2.92E-30	2.33E-29
hsa-mir-4773-1	3.717540957	3.22E-27	2.23E-26
hsa-mir-4773-2	3.781228894	6.51E-27	4.46E-26
hsa-mir-4652	3.254741969	1.27E-19	6.09E-19
hsa-mir-1293	3.963710459	9.21E-18	3.98E-17
hsa-mir-875	4.236303576	1.30E-14	4.78E-14
hsa-mir-3591	2.191496424	1.88E-13	6.67E-13
hsa-mir-374c	2.832071207	1.81E-12	5.93E-12

FC: Fold change, FDR: False discovery rate.

### Construction of the ceRNA network in KIRC

**Fig 2:**
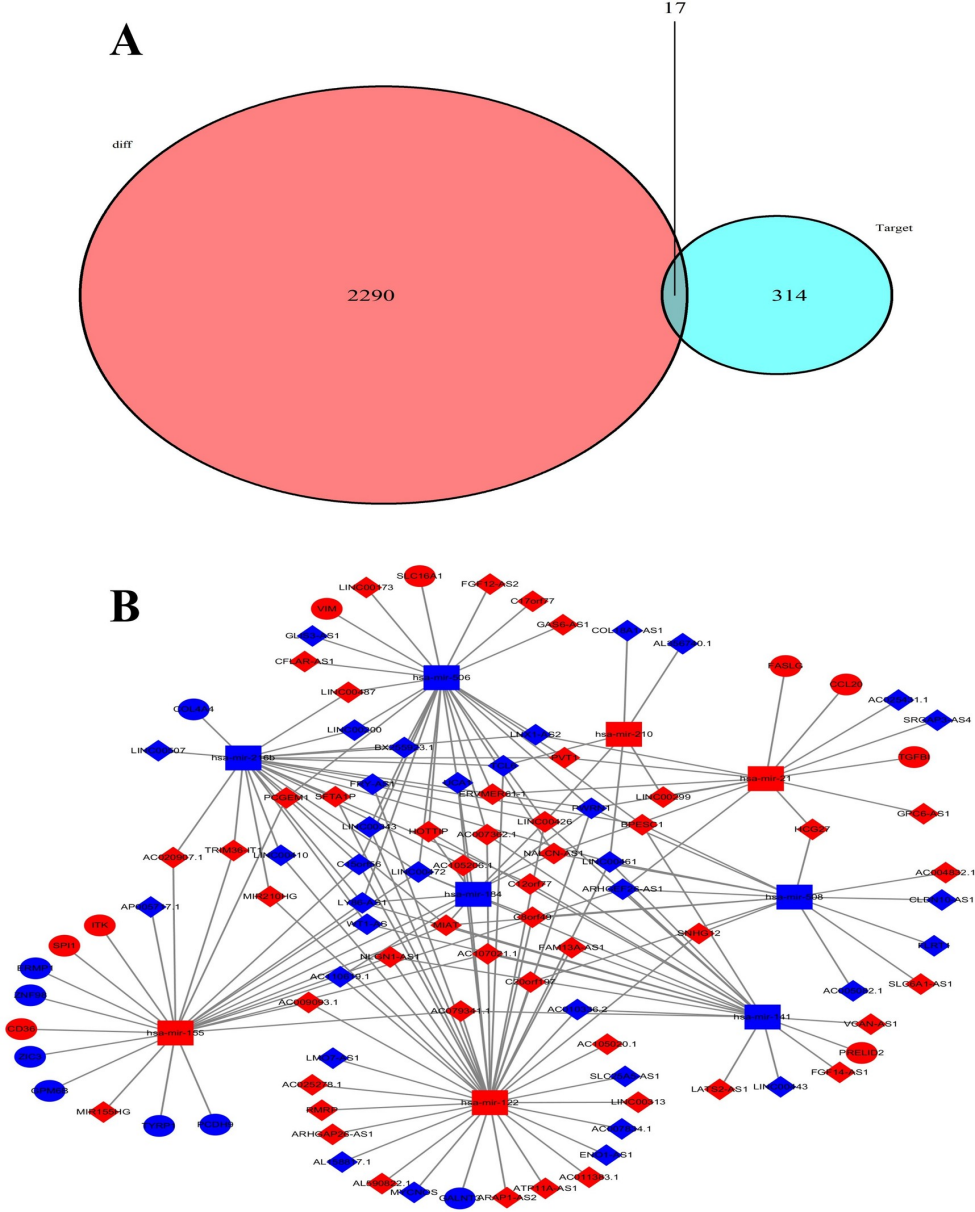
Venn diagram of mRNA and ceRNA networks in KIRC. (a) The intersection of the DEmRNAs and the target mRNAs constituted the Venn diagram. (b) The ceRNA network was composed of DElncRNA, DEmiRNA, and DEmRNA. The diamond was lncRNA, the rectangle was miRNA, and the ellipse was mRNA. Red indicated upregulation, and blue indicated downregulation.

**Fig 3:**
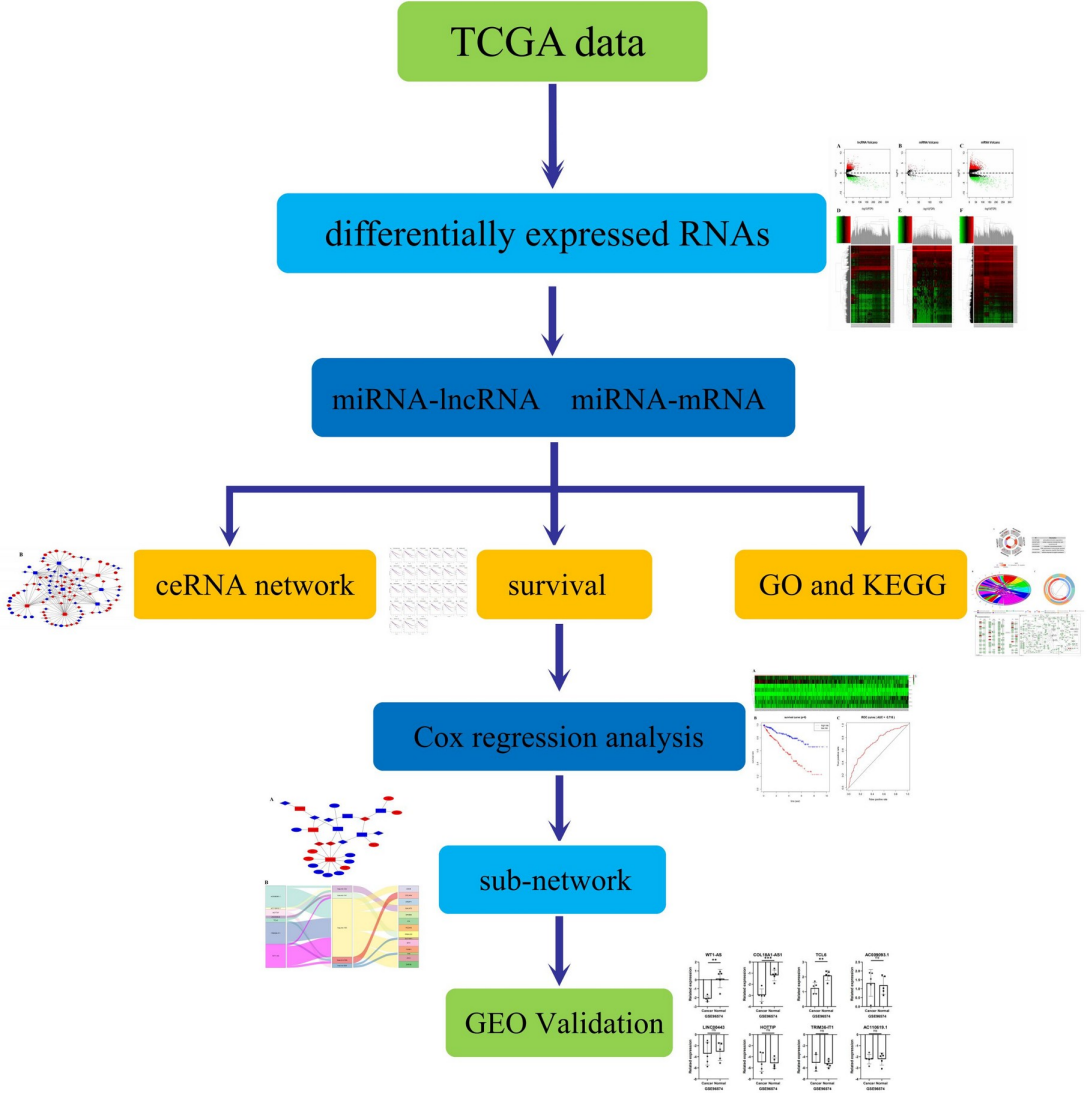
Flow chart of the entire construction process in KIRC. The screening criteria for DEmRNAs, DEmiRNAs and DElncRNAs were |log_2_FC| > 2 and P-value < 0.01. The lncRNA-miRNA interaction was predicted through the miRcode database. lncRNAs unrelated to DEmiRNAs were deleted. The mRNAs targeted by miRNA were predicted using miRTarBase miRDB and TargetScan 3 databases. mRNAs unrelated to DEmRNAs were deleted. The ceRNA network was completed.

To better explore the interaction between differentially expressed RNA in KIRC, we constructed a lncRNA-mediated ceRNA regulatory network to analyze the interaction between them visually. First, We used the miRCode database and paired 1456 DElncRNAs with Perl software to screen out 161 DElncRNA and miRNA relationship pairs. We screened 9 out of 54 DEmiRNAs to interact with 81 DElncRNAs and used the starBase database to convert the miRNA format. Table 5 showed the relationship between lncRNA and miRNA in patients with KIRC. All nine possible target mRNAs of DEmiRNA were screened using Perl software based on three miRNA databases. According to the specified screening conditions, a total of 337 potential mRNA targets were found. Each target appeared in three different miRNA databases. The representative relationship between miRNA and mRNA in patients with KIRC was shown in Table 6. Then we used the “VennDiagrams” package of R software to hybridize these mRNA targets with 2307 identified DEmRNAs to select 16 cross-over genes in KIRC (Fig 2A). As an open-source software platform, Cytoscape is widely used to integrate any type of attribute data to analyze complex network structures visually. We constructed a KIRC ceRNA regulatory network mediated by lncRNA, including 81 DElncRNAs, nine DEmiRNAs, and 17 DEmRNAs. Furthermore, we used Cytoscape to analyze and process the network visually (Fig 2B). The content of the entire ceRNA network included 107 nodes and 178 edges. The flow chart of the entire construction process is shown in Fig 3.

**Table 5:** Representative relationships between lncRNAs and miRNAs in patients with kidney renal clear cell carcinoma.

miRNA	lncRNA
hsa-mir-122	C20orf197,C15orf56,AC105206.1,AC007362.1,LINC00313,TCL6, AC009093.1,SNHG12,RMRP,AL590822.1,AC105020.1,AC025278.1,UCA1, AC010336.2,SLC25A5-AS1,SFTA1P,ARHGAP26-AS1,LINC00343, AC079341.1,NLGN1-AS1,LMO7-AS1,ENO1-AS1,LINC00410,ATP11A-AS1,MYCNOS,FRY-AS1,LINC00426,AC011383.1,AL158817.1,AC007834.1, ARAP1-AS2,LINC00461,MIR210HG,FAM13A-AS1,C8orf49,AC110619.1, PWRN1
hsa-mir-141	AC007362.1,WT1-AS,AC010336.2,LY86-AS1,MIAT,C12orf77,AC079341.1,NLGN1-AS1,LINC00443,AC107021.1,BPESC1,LINC00472,LATS2-AS1, FGF14-AS1,LINC00426,ARHGEF26-AS1,HOTTIP,LINC00461, FAM13A-AS1,VCAN-AS1
hsa-mir-155	C15orf56,AC020907.1,WT1-AS,AC009093.1,LY86-AS1,MIAT,AC079341.1,PCGEM1,NLGN1-AS1,AC107021.1,NALCN-AS1,LINC00472,MIR155HG,ARHGEF26-AS1,AP005717.1,TRIM36-IT1
hsa-mir-184	UCA1,LY86-AS1,MIAT,LINC00472,LINC00426,HOTTIP,C8orf49, AC110619.1,PWRN1
hsa-mir-21	HCG27,ERVMER61-1,NALCN-AS1,SRGAP3-AS4,GPC6-AS1,LINC00299, ARHGEF26-AS1,AC025431.1,LNX1-AS2,PVT1,PWRN1
hsa-mir-210	COL18A1-AS1,TCL6,AL356740.1,LINC00299,LINC00426,LINC00461
hsa-mir-216b	C15orf56,AC105206.1,AC020907.1,WT1-AS,TCL6,BX255923.1, LINC00487,LY86-AS1,SFTA1P,MIAT,C12orf77,LINC00200,LINC00410, BPESC1,LINC00472,LINC00426,LINC00461,MIR210HG,LNX1-AS2,PVT1, TRIM36-IT1,LINC00507,PWRN1
hsa-mir-506	C15orf56,AC007362.1,C17orf77,BX255923.1,LINC00173,LINC00487, UCA1,LY86-AS1,CFLAR-AS1,LINC00343,PCGEM1,LINC00200,FGF12-AS2, ERVMER61-1,BPESC1,NALCN-AS1,LINC00472,GAS6-AS1,GLIS3-AS1, LINC00426,HOTTIP,LNX1-AS2,PVT1,PWRN1
hsa-mir-508	FLRT1,C20orf197,SNHG12,AC004832.1,HCG27,AC005082.1,CLDN10-AS1,MIAT,SLC6A1-AS1,BPESC1,NALCN-AS1,ARHGEF26-AS1,LINC00461, C8orf49,PWRN1

**Table 6:** Representative relationships between miRNAs and mRNAs in patients with kidney renal clear cell carcinoma.

miRNA	mRNA
hsa-mir-122	GALNT3
hsa-mir-141	PRELID2
hsa-mir-155	ERMP1,PCDH9,TYRP1,SPI1,GPM6B,ITK,CD36,ZNF98,ZIC3
hsa-mir-21	FASLG,TGFBI,CCL20
hsa-mir-216b	COL4A4
hsa-mir-506	VIM,SLC16A1

### Screening of survival-related RNA in ceRNA network of KIRC

**Fig 4:**
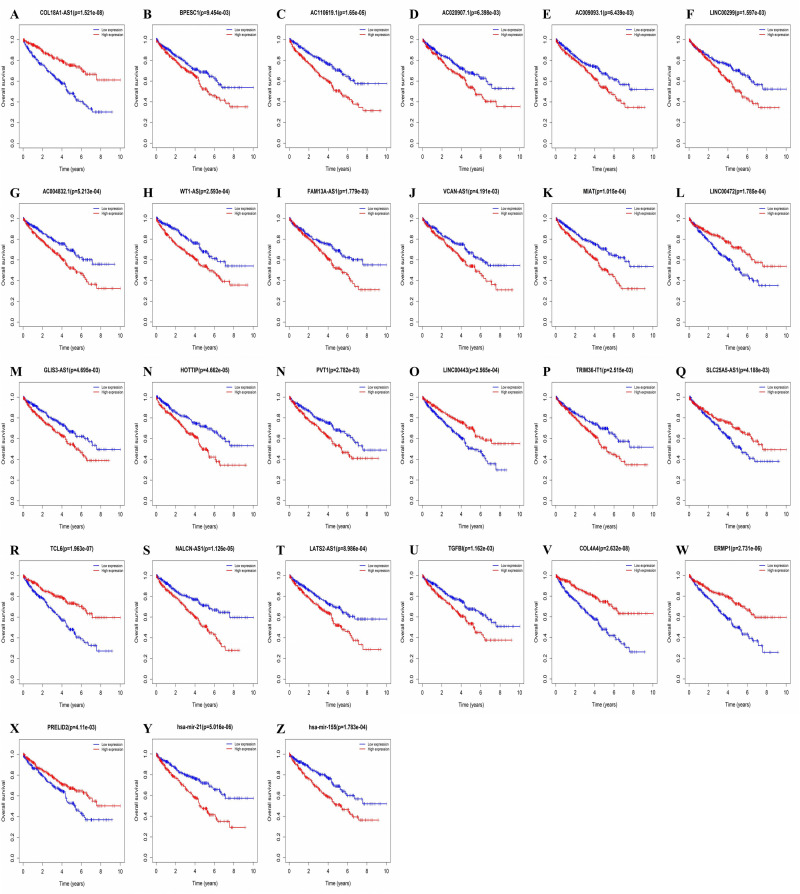
Kaplan–Meier curve analysis and overall survival rate of DElncRNA, DEmRNA, DEmiRNA in KIRC. (a-z) Represented COL18A1-AS1, BPESC1, AC110619.1, AC020907.1, AC009093.1, LINC00299, AC004832.1, WT1-AS, FAM13A-AS1, VCAN-AS1, MIAT, LINC00472, GLIS3-AS1, HOTTIP, PVT1, LINC00443, TRIM36-IT1, SLC25A5-AS1, TCL6, NALCN-AS1, TGFBI, COL4A4, ERMP1, PRELID2, hsa-mir-21, hsa-mir-155.

To find lncRNAs, miRNAs, and mRNAs related to prognosis, we used the “Survival” package to evaluate their relationship with the overall survival rate of patients with KIRC. We once again downloaded the clinical data of patients with KIRC from the TCGA database for survival analysis. The results showed that 21 of the 81 DElncRNAs (COL18A1-AS1, BPESC1, AC110619.1, AC020907.1, AC009093.1, LINC00299, AC004832.1, WT1-AS, FAM13A-AS1, VCAN-AS1, MIAT, LINC00472, GLIS3-AS1, HOTTIP, PVT1, LINC00443, TRIM36-IT1, SLC25A5-AS1, TCL6, and NALCN-AS1) were related to overall survival (Fig 4A–4T). Of the 49 DEmRNAs, a total of four (TGFBI, COL4A4, ERMP1, and PRELID2) were confirmed to be related to the overall KIRC survival (Fig 4U–4X). The analysis showed that two DEmiRNAs (hsa-mir-21 and hsa-mir-155) were related to the overall KIRC survival (Fig 4Y and 4Z). The threshold for all survival analyses was set to P-value < 0.01.

### Functional enrichment and KEGG analysis of differentially expressed mRNA in KIRC

**Fig 5:**
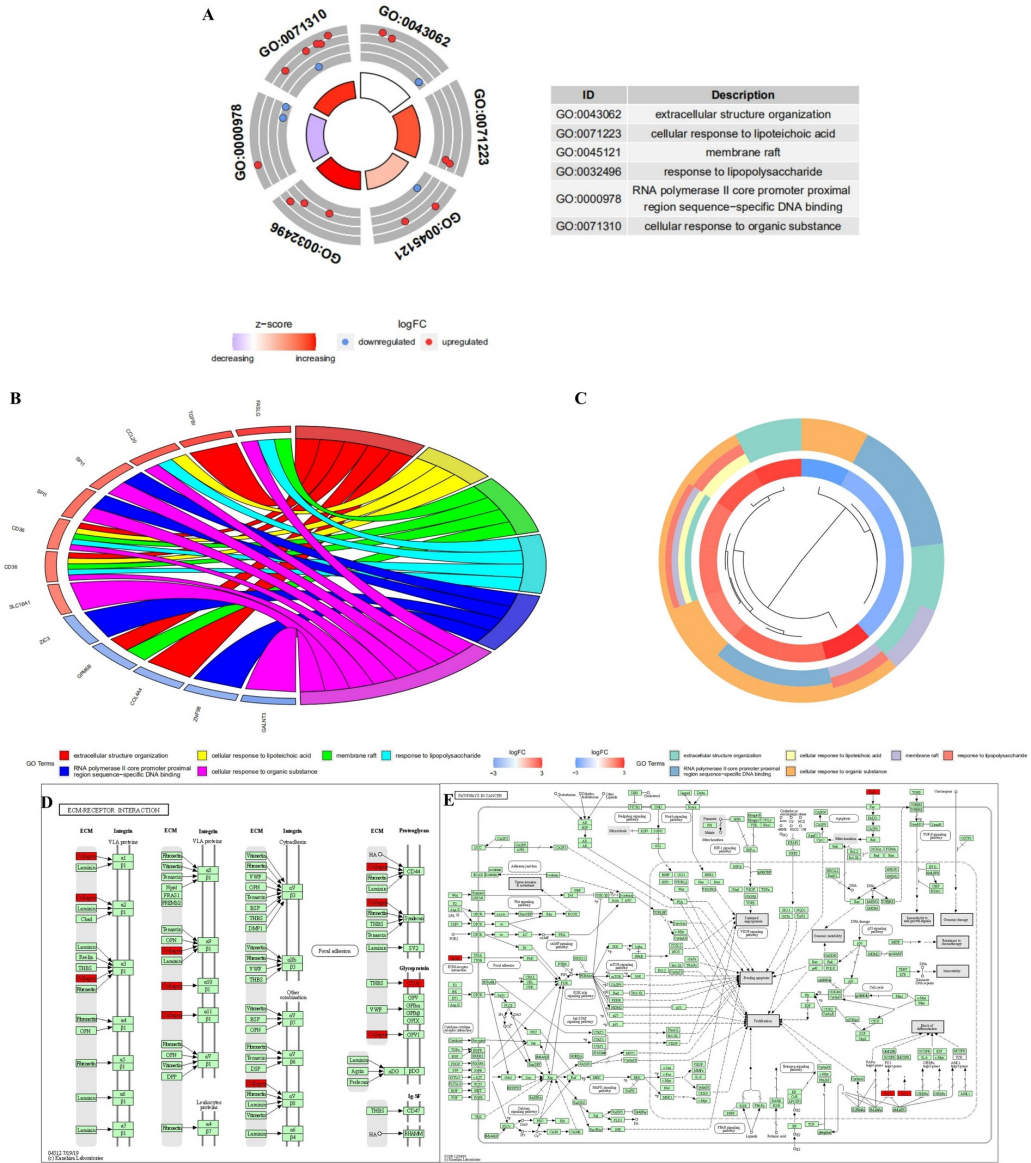
Functional enrichment and KEGG analysis of DEmRNAs related to lncRNA in KIRC. (a-c) The GOCircle, GOChord, and GOCluster diagram of DEmRNAs in KIRC. (d and e) The expression pattern of “ECM-receptor interaction” and “pathways in cancer”.

We performed GO and KEGG analyses using the DAVID database and the KOBAS3.0 online tool to clarify the biological function of DEmRNAs related to lncRNAs. The analysis showed that there were six GO projects in total (P<0.05) and seven KEGG pathways (corrected P <0.05; Tables 7 and 8). In detail, six important GO terms were enriched, suggesting that these DEmRNAs played an essential role in tumors (Fig 5A). Fig 5B shows the interaction between statistically different mRNAs and their related GO terms. Fig 5C shows a cluster analysis of statistically significant mRNA and related GO. KEGG analysis suggested that the two most important were the “ECM-receptor interaction” and “Pathways in cancer” pathways, indicating that these DEmRNAs had an essential role in KIRC (Fig 5E and 5F).

**Table 7:** GO terms of DEmRNA in kidney renal clear cell carcinoma.

Category	ID	Term	Genes	P
BP	GO:0043062	extracellular structure organization	COL4A4, CD36, TGFBI, GPM6B	0.003721138
BP	GO:0071223	cellular response to lipoteichoic acid	CD36, CCL20	0.008617542
CC	GO:0045121	membrane raft	CD36, FASLG, GPM6B	0.032301155
BP	GO:0032496	response to lipopolysaccharide	CD36, CCL20, FASLG	0.033279982
MF	GO:0000978	RNA polymerase II core promoter proximal region sequence-specific DNA binding	SPI1, ZNF98, ZIC3	0.045468359
BP	GO:0071310	cellular response to organic substance	GALNT3, SLC16A1, CD36, CCL20, SPI1, FASLG	0.048776595

BP: Biological process, CC: Cellular Component, MF: Molecular Function.

**Table 8:** KEGG pathway of DEmRNA in kidney renal clear cell carcinoma.

ID	Term	Genes	Corrected P-Value
hsa04512	ECM-receptor interaction	COL4A4,CD36	0.018021432
hsa05200	Pathways in cancer	SPI1,COL4A4,FASLG	0.022038886
hsa04062	Chemokine signaling pathway	CCL20,ITK	0.032483893
hsa04060	Cytokine-cytokine receptor interaction	CCL20,FASLG	0.033020964
hsa05165	Human papillomavirus infection	COL4A4,FASLG	0.033605855
hsa04151	PI3K-Akt signaling pathway	COL4A4,FASLG	0.03384518
hsa05168	Herpes simplex virus 1 infection	FASLG,ZNF98	0.045381132

### Screening of five important lncRNAs related to prognosis inpatients with KIRC

First, we used Perl software to perform univariate Cox regression analysis, a total of 14 lncRNAs were screened out (p <0.001). Next, the "survival" software package of R software was used to perform the next step of multiple Cox regression analysis on the selected lncRNA. As a result, eight lncRNAs were screened out to build a prediction model. The linear combination of the expression of eight lncRNAs was used to build a predictive model (Table 9).


$$\begin{array}{c} riskscore=\left[0.08275\times WT1AS\left(expression\right)\right]+\left[-0.18301\times COL18A1AS1\left(expression\right)\right]\\ +\left[-0.10542\times TCL6\left(expression\right)\right]+\left[0.10326\times AC009093.1\left(expression\right)\right]\\ +\left[-0.32305\times LINC00443\left(expression\right)\right]+\left[0.09631\times HOTTIP\left(expression\right)\right]\\ +\left[0.11648\times TRIM36IT1\left(expression\right)\right]+\left[0.10132\times AC110619.1\left(expression\right)\right]. \end{array}$$

**Table 9:** Multivariate cox regression analysis was performed on eight prognostic lncRNAs of patients with KIRC.

lncRNA	coef	exp(coef)	se(coef)	z	p
WT1-AS	0.08275	1.08628	0.03851	2.149	0.03164
COL18A1-AS1	-0.18301	0.83276	0.05843	-3.132	0.001736
TCL6	-0.10542	0.89995	0.03475	-3.034	0.002416
AC009093.1	0.10326	1.10878	0.06151	1.679	0.093195
LINC00443	-0.32305	0.72394	0.08576	-3.767	0.000165
HOTTIP	0.09631	1.1011	0.05117	1.882	0.059799
TRIM36-IT1	0.11648	1.12353	0.07629	1.527	0.126802
AC110619.1	0.10132	1.10663	0.05841	1.735	0.082796

coef: coefficient, exp: Exponential, se: Standard Error.

### Risk groupings and ROC analysis

**Fig 6:**
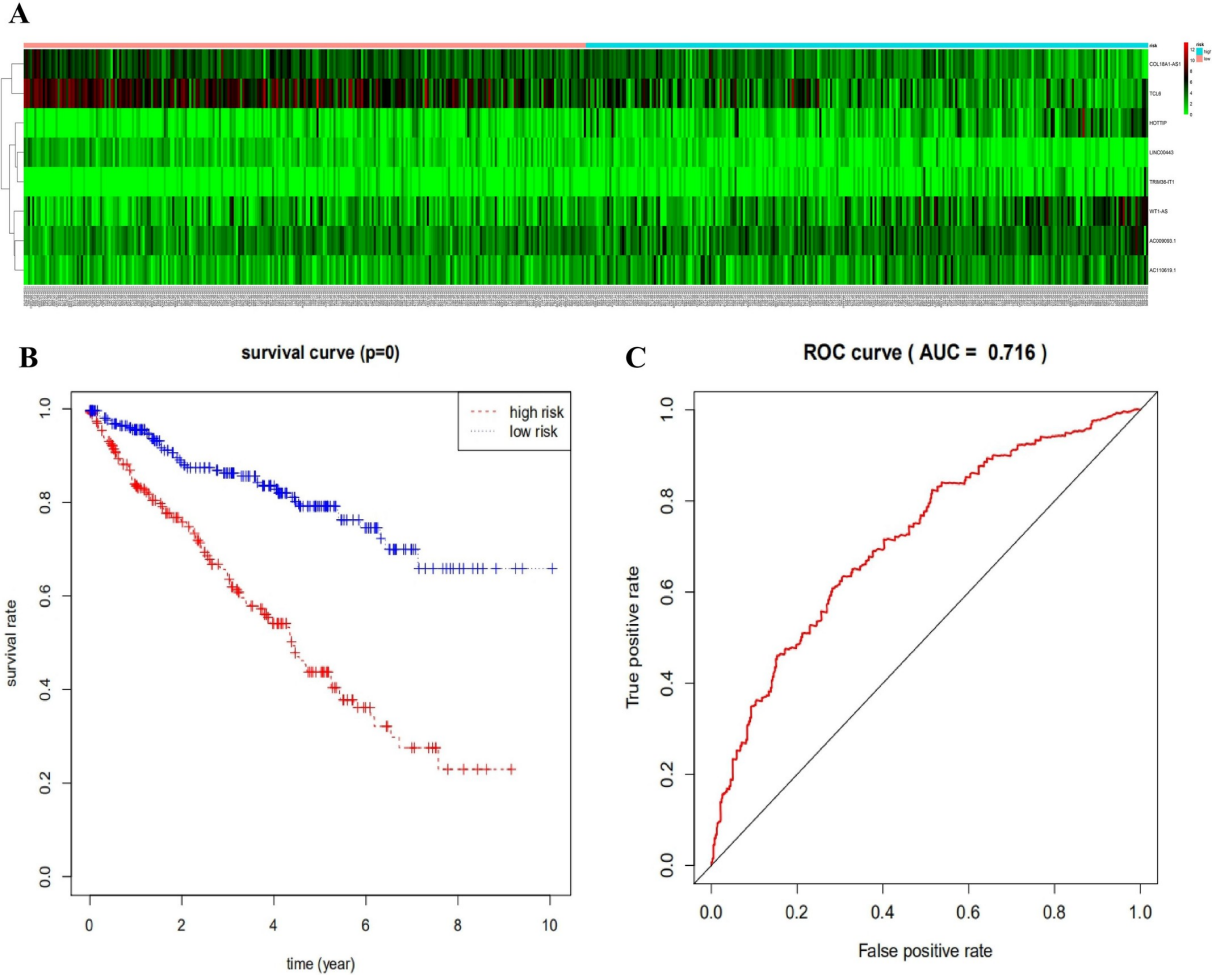
The prognosis of eight lncRNAs in patients with KIRC. (a). Risk heat map based on eight lncRNAs from 530 LUSC patients. (b). Kaplan-Meier curve analysis was performed on the overall survival rate of patients with KIRC using eight lncRNAs. (c). ROC curve analysis was performed on the eight prognosis lncRNAs. AUC: Area Under ROC Curve, ROC curve: Receiver operating characteristic curve.

The "pheatmap" software package of R software was used to visualize the expression of eight lncRNAs (WT1-AS, COL18A1-AS1, TCL6, AC009093.1, LINC00443, HOTTIP, TRIM36-IT1, AC110619.1; Fig 6A). Based on the median risk, we divided a total of 530 complete survival information samples into two groups, including a high-risk group (n = 265) and a low-risk group (n = 265). Next, we used Kaplan–Meier curves in conjunction with log-rank statistical tests for survival analysis. The “survival” package was used for survival analysis. The results showed that there were differences between the two groups, and the low-risk group was significantly better (Fig 6B). The ROC curve analysis was conducted to test the effect of eight lncRNAs on the overall survival rate of KIRC (Fig 6C).

### Prognosis-related DElncRNA-mediated ceRNA in sub-network

**Fig 7:**
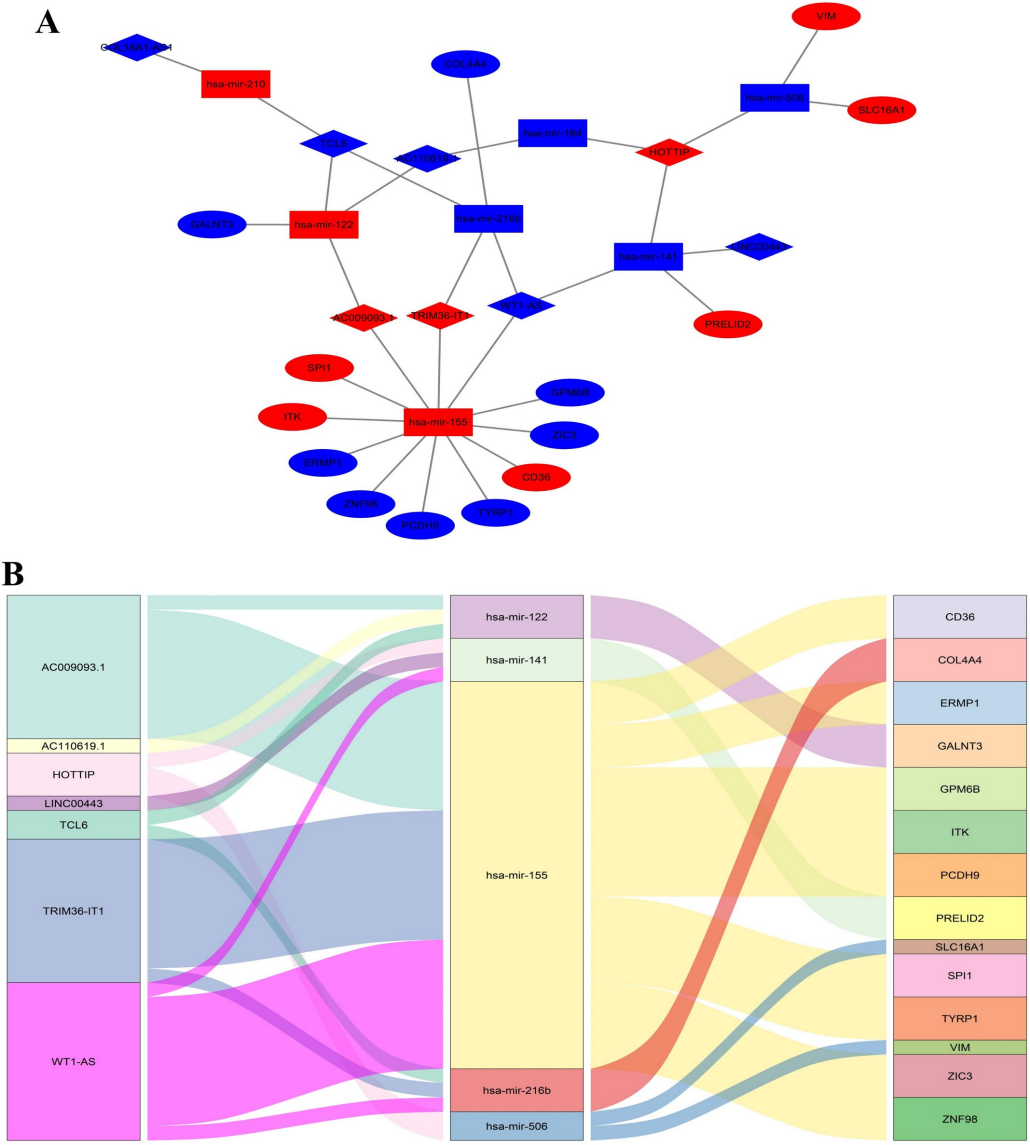
The ceRNA sub-network of the DElncRNA-mediated ceRNA network. (a) The ceRNA sub-network of eight lncRNAs. The diamond was lncRNA, the rectangle was miRNA, and the ellipse was mRNA. Red indicated upregulation, and blue indicated downregulation. (b) The constructed snakey diagram. All involved RNAs were associated with prognosis.

To better analyze the interaction of RNAs in KIRC, we used Cytoscape to analyze the ceRNA network mediated by prognosis-related DelncRNA. As shown in Fig 7A, only eight DElncRNAs, seven DEmiRNAs, and 14 DEmRNAs were shown in the DElncRNA-mediated ceRNA sub-network. This newly reconstructed sub-network consisted of 29 nodes and 31 edges. We used the Sankey diagram constructed by the “ggalluival” package of R software to visually analyze ceRNA regulatory sub-network mediated by eight lncRNAs related to prognosis (Fig 7B).

### Validation of independent data sets

**Fig 8:**
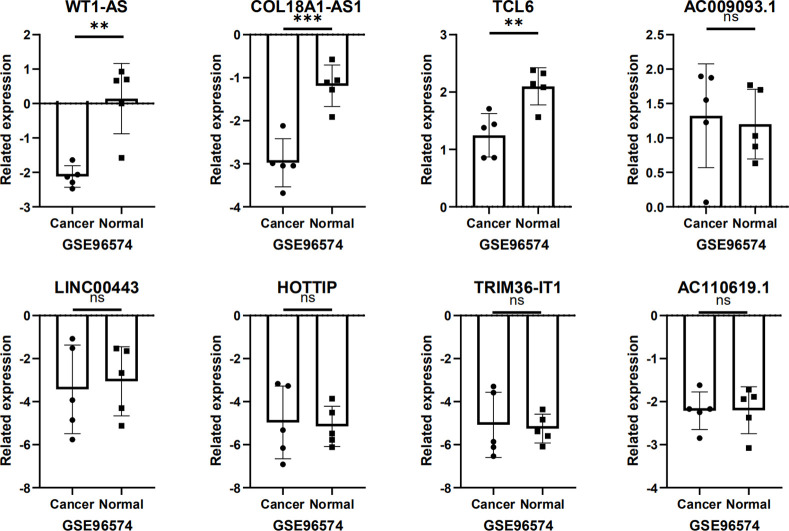
In the independent dataset GSE96574, the eight lncRNAs selected were verified. NS: no significance.

We validated these eight lncRNAs using the independent dataset GSE96574 in the GEO database. Among them, WT1-AS, COL18A1-AS1, and TCL6 had statistical differences. The results showed that there were three statistical differences (Fig 8).

## Discussion

The recent increase in patients with KIRC highlights the urgent need for more effective prognostic biomarkers to predict the prognosis. lncRNA-related research has attracted extensive attention in the field of cancer. Several studies have shown that lncRNAs related to cancer can be used as a biomarker for the diagnosis or prediction of cancer and plays a vital role in cancer treatment. Although the molecular mechanisms of KIRC protein-coding genes or miRNAs have been studied, little is known about lncRNAs. Few have predicted the prognosis of lncRNA in KIRC and the construction of the ceRNA network. There were no reliable specific lncRNA KIRC biomarkers. Moreover, there remained a lack of RNA sequencing data and analysis related to lncRNA profiles in KIRC. Qin Li et al. studied three hub genes of VHL, SETD2, and TRIP11 from KIRC as possible therapeutic targets and established and verified related ceRNA networks and verified it [38]. Few studies explored lncRNA biomarkers and constructed KIRC-releated ceRNA networks [39].

CeRNAs reveal a new mechanism of RNA interaction and reflects the interactions of coding RNA and non-coding RNA. CeRNAs are regarded as balances; when disrupted, they lead to the disturbance of life activities and the occurrence of diseases, especially in tumor diseases [40]. Many ceRNA network imbalances between these non-coding RNAs play a fundamental role in establishing signs of cancer development. The lncRNA OSER1-AS1promotes the deterioration of non-small cell lung cancer by sponging microRNA‑433‑3p to further increase Smad2 expression [41]. The lncRNA LINC00689 causes miR-526b-3p to sponge and leads to the upregulation of ADAM9 to promote the progression of gastric cancer [42]. These findings open a new window for a better understanding of cancer’s hidden aspects and also highlight novel biomarkers and potentially effective therapeutic targets for cancer. Xiaolong Qi et al. proposed that ceRNA may provide new biomarkers and potentially effective therapeutic targets for breast cancer [43]. Juan Xu et al. constructed mRNA-related ceRNAs in 20 major cancer types and further revealed conserved and reconnected network ceRNA hubs in each cancer. These highly competitive interactions that constitute a conservative and cancer-specific module [44]. Chu Y et al. also found that lncRNA perturbed TLR signaling network performance can serve as a new lncRNA prognostic biomarker in colorectal cancer [45].

Increasing evidence shows that lncRNAs are key members in ceRNA and regulate other RNAs in the ceRNA network [46]. An effective way to study the potential of lncRNAs is by establishing a relationship between the lncRNA of interest and miRNA/mRNA. CASC9 promotes the progression of non-small cell lung cancer through the miR-335-3p/S100A14 axis [47]. The lncRNA SNHG3 promotes the progression of bladder cancer via the miR-515-5p/GINS2 axis [48]. The lnc-HSD17B11-1:1 was found to promote colorectal cancer progression through the lnc-HSD17B11-1:1/miR-338-3p/MACC1 axis [49]. The lncRNA FAM83H-AS1 promotes the progression of esophageal squamous cell carcinoma through miR-10a-5p/Girdin axis [50]. The lncRNA PVT1 also promotes gastric cancer migration by acting as a ceRNA for miR-30a and regulating snails [51]. The lncRNA SNHG17 also promotes the progression of glioma cells by regulating the miR-23b-3p/ZHX1 axis [52]. The STAT3-mediated lncRNA HOXD-AS1 is upregulated by ceRNA and subsequently promotes the metastasis of liver cancer by regulating SOX4 [53]. The lncRNA FAL1 also promotes the proliferation and metastasis of hepatocellular carcinoma cells by acting as a ceRNA of miR-1236 [54]. Therefore, looking for lncRNAs related to prognosis and understanding its regulation mechanism and functional role is vital for KIRC diagnosis and treatment.

It is increasingly challenging increasingly difficult to detect potential human lncRNA-disease associations from these huge biological data sets using traditional biological experimental methods. Therefore, it is important to develop new and effective calculation methods to predict potential human lncRNA diseases. Several experimental methods and computational models have been designed and implemented to identify novel ncRNA-disease associations [55, 56]. For example, some researchers used the principles of machine learning to construct a new type of interpretable analysis to analyze potential biomarkers of tumorigenesis in non-small cell lung cancer. This improves our understanding of the nature of the disease and helps discover more effective diagnosis, prognosis and treatment methods for various cancer types and stages [57]. A semi-supervised interactome network-based method was recently used to explore and predict potential interactions between lncRNA and miRNA. The model will help predict the interaction between lncRNA and miRNA, and successfully demonstrated superiority and good generalizability [58]. In another study, researchers used a combination of incremental principal component analysis and random forest algorithm. By integrating multiple similarity matrices, we proposed a new algorithm based on integrated machine learning technology to predict associated lncRNA diseases [59]. A new type of induction matrix completion model for MiRNA-disease association prediction has been proposed [60, 61]. The study found that the method of Laplacian Regularized Least Squares for LncRNA-Disease Association (LRLSLDA) was further developed in the semisupervised learning framework. It will benefit biomarker identification and drug development. It can be expected that LRLSLDA can become an effective and important biological tool for biomedical research [62]. These studies show that the methods and techniques of computational biology are playing an increasingly important role in medicine and biology [63, 64]. In the future, we need to use more computational biology techniques to research KIRC to find more suitable and effective prediction and analysis models. We searched the KIRC transcriptome and clinical data from the TCGA database. Our research used the “edgeR” package to obtain DEmRNA, DEmiRNA, and DElncRNA. We used the miRcode database to screen the target miRNA corresponding to DElncRNA. Next, we searched and determined the target mRNA of DEmiRNA together across three databases. Finally, the ceRNA network related to lncRNA was established. The new ceRNA network was composed of 81 DElncRNAs, nine DEmiRNAs, and 17 DEmRNAs. This network included 107 nodes and 178 edges. Subsequently, we used survival analysis to find RNA associated with all prognoses. We then analyzed DEmRNAs using the GO and KEGG pathways.

We often need ways to enrich related genes to study the relationship between them, with the increasing availability of useful online tools. The two important online tools are DAVID and KOABS; they can perform functional enrichment analysis and pathway analyses online to study the mechanism of downstream pathways and potential targets [65, 66]. The tools and data processing methods constructed by computer biology have played a huge role in promoting the huge data of medicine and biology and helped cure diseases. GO functional enrichment and KEGG pathway analyses are widely used to evaluate the rich biological functions in multiple coding genes. Our GO analysis showed that most of the DEmRNAs were enriched in extracellular structure organization, cellular response to lipoteichoic acid, membrane raft, response to lipopolysaccharide, RNA polymerase II core promoter proximal region sequence-specific DNA binding, and cellular response to an organic substance. KEGG pathway analysis also showed that most of the DEmRNAs were enriched in ECM-receptor interaction, cancer pathways, chemokine signaling pathway, cytokine-cytokine receptor interaction, human papillomavirus infection, PI3K-Akt signaling pathway, and herpes simplex virus 1 infection.

Due to the heterogeneity between patients, traditional prognostic systems are often incapable of making accurate predictions of risk grouping and clinical outcomes. Therefore, several studies have been constructed new prediction models to carry out the survival expectation of KIRC. Furthermore, we analyzed and screened some DElncRNAs, selected eight lncRNAs related to prognosis, and constructed ROC curves and COX regression models. These eight lncRNAs were screened to construct a predictive model for early diagnosis and prognosis of KIRC. Survival analysis based on the linear combination of 8-lncRNA signals showed that the survival rate of high-risk groups was lower than that of low-risk groups. Therefore, these eight lncRNAs (WT1-AS, COL18A1-AS1, TCL6, AC009093.1, LINC00443, HOTTIP, TRIM36-IT1, and AC110619.1) have a very significant connection with the prognosis of KIRC. Finally, we verified these eight lncRNAs with independent datasets and found that expression of WT1-AS, COL18A1-AS1, and TCL6 were statistically different. However, as the sample size of the independent data set in GEO was small, it did not mean that the other lncRNAs were not statistically different. In the future, a larger amount of sequencing data and experiments are needed to verify these targets.

WT1-AS inhibits the proliferation of cervical squamous cell carcinoma cells by regulating p53 [67]. The overexpression of lncRNA WT1-AS affects non-small cell lung cancer cells by regulating TGF-β1 [68]. WT1-AS promotes hepatoma cell apoptosis by downregulating WT1 [69]. TCL6 inhibits the development of lncRNA in cancer, and in hepatocellular carcinoma directly regulated the PI3K / AKT signaling pathway by binding to miR-106a-5p to affect cancer development [70]. The mechanism and role of several other lncRNAs in cancer remain unclear. Future studies of these lncRNAs should focus on KIRC prognosis-related biomarkers. Our research screened out potential targets for KIRC using bioinformatics and computational biology methods construct a Cox regression analysis model. This will help in the diagnosis and treatment of patients with KIRC going forward, and guide the construction of predictive models and identification of potential targets in other diseases. The TCGA database was the key to analyzing prognostic biomarkers; all of our data were obtained from here. The primary method we used involved bioinformatics, seemingly the most promising way to explore gene and protein pathway and network interactions. However, the function and network of lncRNAs were very complicated. More clinical trials are required to verify our findings in the future. Finally, the biological functions of the eight lncRNAs with KIRC prognosis should be verified through large-scale experiments.

## Conclusion

We constructed ceRNA networks using transcriptome sequencing data in the TCGA database and screened out DElncRNsA, DEmiRNAs, and DEmRNAs, which may be related to KIRC pathogenesis. The eight lncRNAs screened in the ceRNA network constructed a predictive model and may play a vital role in the diagnosis and prognosis of patients with KIRC. And three of them by independent samples proved to be meaningful.

## Supporting information

S1 File Original material. (ZIP)

S2 File Source code. (ZIP)
